# Introduction to Endo-ERN–scope and mission

**DOI:** 10.1007/s12020-020-02602-z

**Published:** 2021-01-15

**Authors:** Alberto M. Pereira, Olaf Hiort

**Affiliations:** 1 Department of Medicine, Division of Endocrinology and Centre for Endocrine Tumors Leiden (CETL), Leiden University Medical Centre, Leiden, the Netherlands; 2 Division of Paediatric Endocrinology and Diabetes, Department of Paediatrics and Adolescent Medicine, University of Lübeck, Lübeck, Germany

**Keywords:** Endo-ERN, Rare disease, Patient perspective, Endocrinology

## Abstract

The official installation of the European Reference Networks in 2017 formed the foundation to improve quality and safety and access to highly specialized health care across the EU for patients affected by rare or low prevalence and complex conditions. The European Reference Network on Rare Endocrine Conditions (Endo-ERN) covers specific expertise from birth to senescence with a specific governance structure characterized by both a pediatric and an adult chair, and equal responsibilities for patient representatives and health care providers. The introduction on the scope and mission of Endo describes the complexity of the Endo-ERN mission and will thrive toward the ultimate aim and mission of the network of reducing health care inequalities across Europe. Specific knowledge and medical expertise of the existing rare endocrine condition is urgently needed, and therefore, raising awareness for Rare Disease Day from the Endo-ERN perspective is imperative.

The EU directive 2011/24 of patient’s rights in cross border health care that was accepted by the European parliament in 2011 formed the foundation for the development of European Reference Networks (ERNs) [1]. ERNs are virtual networks of health care providers (HCPs) aiming at improving quality and safety and access to highly specialized health care across the EU for patients affected by rare or low prevalence and complex conditions. The added value at the European level is evident as it facilitates the need for cooperation, as scarcity of knowledge, need for education, complexity/high costs, and effective use of resources that are hallmarks of rare and complex conditions. ERNs have to be broad thematic networks to ensure that all patients with rare and/or complex conditions have ‘a home’. In addition, the networks should be patient centered, both in terms of the organization of the care, and in terms of governance/expertise, preferably providing expert care throughout the entire lifespan [2]. In 2017, 24 ERNs, including Endo-ERN, were installed linking >300 hospitals and 900 expert centers, and including 24 European Patient Advocacy Groups (ePAG) of >170 patient organizations & <1000 ePAG member organizations.

The European Reference Network on Rare Endocrine Conditions (Endo-ERN) was initiated by clinical committee members of both European endocrine societies: the European Society of Pediatric Endocrinology (ESPE) and the European Society of (adult) Endocrinology (ESE) that identified and nominated a pediatric and an adult chair as well as steering committee members who developed and decided upon the coordination and governance. On the landscape for rare endocrine conditions Endo-ERN was divided into eight organ- and physiology-based main thematic disease groups (MTGs: Adrenal, Disorders of calcium & phosphate homeostasis, Genetic disorders of glucose & insulin homeostasis, Genetic endocrine tumor syndromes, Growth & genetic obesity syndromes, Pituitary, Sex development & maturation, and Thyroid) which were included in five Work Packages (WPs: Education & training, E-health & ICT, Research & Science, Quality Control & Patient View, Diagnostics & Laboratory analysis).

Endo-ERN, at present, is the largest ERN, representing 86 HCPs from 26 countries. Patient involvement is crucial to all ERNs, and Endo-ERN already integrated patient representation during the construction phase of the network, with equal responsibilities for patient representatives and HCPs [3]. Each MTG and WP is co-chaired by 2 endocrinologists, specialized in pediatric and adult endocrinology, respectively, and at least one patient representative. All patient representatives are endorsed by their national organizations and have been approved by Eurordis, the European umbrella organization for rare disease patient organizations, and have officially been installed as European Patient Advocacy Group (ePAG) representative [4]. These representatives ensure the presence of the patients’ voice within the Network.

This special and unique supplemental issue of ‘Endocrine’ has been compiled by Endo-ERN experts and encompasses a large array of specific features of rare endocrine conditions, covering specific activities carried out in the different work packages and main thematic groups of the network, and range from mini-reviews of a specific condition to manuscripts describing original novel data, including data on the use of virtual consultations, and on the educational and research needs from the patient’s perspective, representing all work packages and main thematic groups. With this, we are confident that the complexity of the Endo-ERN mission is well described and we will thrive towards the ultimate aim of reducing health care inequalities across Europe.

Written and compiled in the midst of the Covid-19 pandemic, raising awareness for Rare Disease Day from the Endo-ERN perspective is quite necessary. As we will see from the manuscripts, the specific knowledge and medical expertise of the existing rare condition was mandatory for battling corona infections in our patients. Furthermore, during the pandemic, the anxiety of chronically ill patients increased and their medical attention could often not be met. With the more specific medical but also societal approaches to Covid-19, it is time to turn again to all medical necessities of our European fellow citizen, so no one is “forgotten”.
